# Flexible Glassy Carbon Multielectrode Array for *In Vivo* Multisite Detection of Tonic and Phasic Dopamine Concentrations

**DOI:** 10.3390/bios12070540

**Published:** 2022-07-20

**Authors:** Elisa Castagnola, Elaine M. Robbins, Bingchen Wu, May Yoon Pwint, Raghav Garg, Tzahi Cohen-Karni, Xinyan Tracy Cui

**Affiliations:** 1 Department of Bioengineering, University of Pittsburgh, Pittsburgh, PA 15260, USA; elc118@pitt.edu (E.C.); emr72@pitt.edu (E.M.R.); biw15@pitt.edu (B.W.); m.pwint@pitt.edu (M.Y.P.); 2 Center for Neural Basis of Cognition, University of Pittsburgh, Pittsburgh, PA 15261, USA; 3 Department of Materials Science and Engineering, Carnegie Mellon University, Pittsburgh, PA 15213, USA; thenamegarg@gmail.com (R.G.); tzahi@andrew.cmu.edu (T.C.-K.); 4 Department of Biomedical Engineering, Carnegie Mellon University, Pittsburgh, PA 15213, USA; 5 McGowan Institute for Regenerative Medicine, University of Pittsburgh, Pittsburgh, PA 15219, USA

**Keywords:** multielectrode array, carbon, PEDOT/CNT, fast-scan cyclic voltammetry, square-wave voltammetry

## Abstract

Dopamine (DA) plays a central role in the modulation of various physiological brain functions, including learning, motivation, reward, and movement control. The DA dynamic occurs over multiple timescales, including fast phasic release, as a result of neuronal firing and slow tonic release, which regulates the phasic firing. Real-time measurements of tonic and phasic DA concentrations in the living brain can shed light on the mechanism of DA dynamics underlying behavioral and psychiatric disorders and on the action of pharmacological treatments targeting DA. Current state-of-the-art *in vivo* DA detection technologies are limited in either spatial or temporal resolution, channel count, longitudinal stability, and ability to measure both phasic and tonic dynamics. We present here an implantable glassy carbon (GC) multielectrode array on a SU-8 flexible substrate for integrated multichannel phasic and tonic measurements of DA concentrations. The GC MEA demonstrated *in vivo* multichannel fast-scan cyclic voltammetry (FSCV) detection of electrically stimulated phasic DA release simultaneously at different locations of the mouse dorsal striatum. Tonic DA measurement was enabled by coating GC electrodes with poly(3,4-ethylenedioxythiophene)/carbon nanotube (PEDOT/CNT) and using optimized square-wave voltammetry (SWV). Implanted PEDOT/CNT-coated MEAs achieved stable detection of tonic DA concentrations for up to 3 weeks in the mouse dorsal striatum. This is the first demonstration of implantable flexible MEA capable of multisite electrochemical sensing of both tonic and phasic DA dynamics *in vivo* with chronic stability.

## 1. Introduction

Dopamine (DA) is an electroactive monoamine that plays a central role in a variety of brain functions, including behavior and cognition [1,2,3,4], reward [5,6], and voluntary movement [7,8,9]. DA release occurs over multiple timescales, including fast phasic release (milliseconds to seconds), caused by neuronal firing in response to stimuli [10,11], and tonic release, slow-changing tonic levels (seconds to minutes [10,12]) that regulate the phasic release through its effect on extracellular DA levels [13]. Deficiency of the dopaminergic system has been implicated in different neurological and psychiatric disorders, including Parkinson’s disease [4,14,15], schizophrenia [16,17], drug abuse [18,19,20], eating disorders [21,22,23], and obsessive-compulsive disorders (OCD) [24,25,26]. In particular, alterations in the coaction of tonic and phasic DA dynamics, the so-called tonic/phasic DA model, have been shown to be strongly implicated in schizophrenia [11,13], addiction [12,27,28], Tourette’s syndrome [29,30], OCD [30], and Parkinson’s disease [31]. Therefore, sensors capable of multimodal measurements of phasic and tonic DA releases from multiple locations of the brain are of fundamental importance for elucidating brain function and improving the diagnosis and pharmacological treatments of these neurological and psychiatric deficits.

For the last 3 decades, fast-scan cyclic voltammetry (FSCV) at carbon-fiber microelectrodes (CFEs) has been considered the gold standard for *in vivo* DA detection [32,33], significantly advancing our understanding of phasic DA dynamics [34,35]. FSCV relies on the direct electron transfer reaction between redox-active molecules and the carbon surface of the electrodes. By sweeping the potential window at fast scan rates, usually 400 V/s for DA, FSCV achieves a subsecond temporal resolution [36,37,38], consistent with the scale of neurotransmitter release at synapses [33,39,40,41]. While FSCV can efficiently measure rapid changes in concentration, i.e., phasic DA release, the necessity for background subtraction limits its ability to monitor tonic dynamic and ambient neurotransmitter levels [42,43].

The most widely adopted *in vivo* sampling technique for tonic DA detection is microdialysis [44,45,46], which must be coupled with an analytical method such as high-performance liquid chromatography (HPLC) or electrochemical techniques [47,48,49], to identify and quantify the neurochemicals in the dialysate. Additionally, it suffers from poor temporal resolution (minutes [50]), preventing direct correlation of tonic and phasic DA concentrations to neural spiking activity [51,52]. Additionally, implantation of large-diameter microdialysis probes (~200–300 μm) creates substantial tissue damage that greatly diminishes extraction efficiency over time [53,54,55] and does not guarantee spatial resolution. Alternatively, several electrochemical methods have been recently developed, including fast-scan controlled-adsorption voltammetry (FSCAV) [56,57], charge-balancing multiple waveform FSCV [39], and convolution-based FSCV [58], for recording tonic DA concentrations in the brain. These measurements have been performed at single CFEs, limited to only one active site per penetrating electrode. To enable multisite sensing, CFE arrays have been fabricated and demonstrated to have excellent phasic DA detection in acute and chronic studies [59,60]. However, their manufacturing process has been semi-manual and does not permit high-density 3D-electrode site arrangement or batch fabrication.

On the other hand, lithographically fabricated multielectrode arrays (MEAs) can be batch produced with high yield and high spatial precision and are routinely used for measuring neurophysiological activity from multiple sites across different depths and widths of the brain with high-quality, single-unit resolution [56,61]. Most MEAs are fabricated by patterning metals on rigid silicon substrates. Because metal electrodes (Au, Pt, Ir) present poor sensitivity towards dopamine when using direct electrochemical detection, they cannot be directly used for DA sensing [62,63]. Furthermore, implantation of stiff MEAs results in the formation of a “kill zone” directly surrounding the implant, characterized by significantly lower neuron density and increased glial encapsulation [64,65,66]. These tissue responses likely compromise both electrophysiological and neurochemical measurements in chronic applications [67,68,69,70]. To reduce the mechanical mismatch-induced tissue response, thin-film polymers, such as polyimide, parylene C, and SU-8, have been used as substrates for flexible MEAs to match the soft nature of the brain and minimize micromotion-induced inflammation [70,71,72]. While these flexible probes along with the subcellular dimension have been shown to seamlessly integrate with the neural tissue and record stable neural signals for months [73,74,75,76], the electrode materials still need to be optimized to enable sensitive and stable electrochemical detection.

Carbon is considered the ideal material for electrochemical sensing [77,78,79] and presents superior electrochemical stability [73,80]. However, only a handful of efforts have been documented in the literature to use carbon as an electrode material in flexible arrays to enable sensing [81,82,83,84,85]. Indeed, the high temperatures required for carbon synthesis are incompatible with polymer substrates. [86,87] Glassy carbon (GC) has only recently been considered for implantable neural interfaces, mainly due to a key advanced technology, developed by the Kassegne lab, that allows for pattern transfer and integration of prepyrolyzed GC electrodes into flexible circuits with metal traces and interconnections [83,88]. The pyrolysis process of SU-8 has been demonstrated to produce carbon structures with a high degree of graphitization [89,90]. GC microelectrodes pyrolyzed this way demonstrated the capability to detect low DA concentrations using FSCV [82,85] and resist electrochemical fouling [85].

In this work, we developed the first implantable carbon-based flexible MEA capable of multisite electrochemical sensing of both tonic and phasic DA dynamics *in vivo*.

First, we fabricated carbon-based MEAs using a previously developed pattern-transfer technique that enable the integration of GC microelectrodes into flexible substrate [88,91]. Here, to bring this technology to the next level, we optimized a high-resolution maskless direct-writing photolithography process to transfer GC MEAs on a thin, flexible SU-8 substrate with significantly reduced form factors to promote tissue integration. Secondly, we incorporated a poly(3,4-ethylenedioxythiophene)/acid-functionalized carbon nanotube (PEDOT/CNT) coating on selected GC microelectrodes to enable direct tonic DA measurement *in vivo* using square-wave voltammetry (SWV), a pulse voltammetry technique designed to directly measure resting analyte concentrations by isolating faradaic current (resulting from redox activity derived from an electroactive analyte) from nonfaradaic charging currents (resulting from the charging of the electric double layer) [92,93]. We previously demonstrated that PEDOT/CNT coating increases the sensitivity for DA SWV detection by a factor of 422, compared to uncoated CFEs, and enables multisite detection of tonic DA in multiple brain regions using metal MEA [93]. Finally, we demonstrate the capability of our MEA for *in vivo* multichannel detection of phasic DA release, using FSCV at GC microelectrodes, and stable multichannel detection of tonic DA concentrations using SWV at PEDOT/CNT-coated microelectrodes, in the mouse dorsal striatum. As a proof of concept, our flexible PEDOT/CNT-coated MEAs achieved detection of tonic DA concentrations for up to 3 weeks, with minimal variations in the DA peak amplitude and electrochemical impedance.

To the best of our knowledge, this is the first flexible device capable of chronic, multichannel measurements of tonic and phasic DA dynamics *in vivo*, providing a powerful tool for neuroscience research.

## 2. Materials and Methods

### 2.1. GC MEA Fabrication

A 4-in Si wafer with a 100 µm thick SiO_2_ layer (University Wafer Inc. Boston, MA, USA) was first cleaned with acetone, isopropanol, and DI water sequentially. The wafer was then dried with a N_2_ spray gun, heated on a hot plate at 150 °C for 5 min, and treated with O_2_ plasma using a reactive ion etcher (RIE, Trion Phantom III LT) for 2 min at 300 mTorr pressure and 150 W power. The cleaned wafer was spin-coated with SU-8 100 (MicroChemicals, Ulm, Germany) at 5000 rpm for 1 min and soft baked at 65 °C for 5 min and 95 °C for 15 min. Then, the wafer was exposed using a direct-writing maskless aligner (MLA, MLA100 Heidelberg Instruments) with a dose of 500 mJ/cm^2^. After exposure, the wafer was first post-baked at 65 °C for 3 min and 95 °C for 5 min, then developed using a SU-8 developer (MicroChemicals) for 5 min and cleaned with isopropanol and DI water. The patterned SU-8 was subsequently hard baked at 200 °C, 180 °C, and 150 °C for 5 min each and allowed to cool down below 95 °C. Pyrolysis of the negative SU-8 resist was performed in a custom-designed chamber. Briefly, the samples were heated to 1000 °C with a temperature ramp-up at a rate of 3.5 °C/min, then maintained at 1000 °C under 15 standard cubic centimeters per minute (sccm) N_2_ (Airgas) at 0.8 Torr for 60 min. The samples were then slowly cooled to room temperature.

After the pyrolysis, the wafer was cleaned with acetone, isopropanol, and DI water sequentially and treated with O_2_ plasma with RIE for 90 s at a pressure of 200 mTorr and 150 W power. The cleaned wafer was then spin-coated with SU-8 2005 (MicroChemicals) at 4000 rpm for 1 min, then soft based at 65 °C for 3 min and 95 °C for 5 min. This first SU-8 layer was patterned, using the MLA with a dose of 300 mJ/cm^2^, to define the bottom insulation layer and to open a connection between the GC electrodes and the metal traces (next step). After a post-bake at 65 °C for 3 min and 95 °C for 5 min, the wafer was developed using the SU-8 developer. Finally, the patterned wafer was cleaned with isopropanol and DI water, hard baked at 200 °C, 180 °C, and 150 °C for 5 min each, and allowed to cool down below 95 °C.

**Figure 1:**
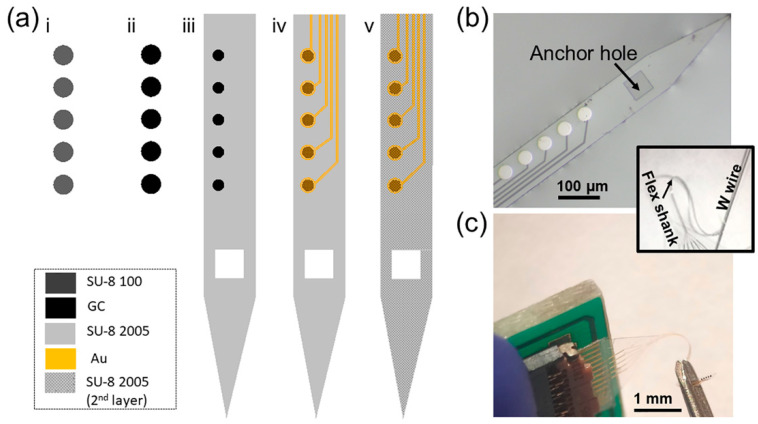
Flexible GC-coated hybrid MEA. (**a**) Schematic for fabrication of the GC MEAs: (**i**) SU-8 100 spin-coating and patterning of electrodes on SiO_2_ wafer; (**ii**) pyrolysis; (**iii**) SU-8 insulation layer spin-coating on top of the GC electrodes and UV exposure to pattern the insulation layer and open a connection between the GC electrodes and the metal traces (next step) and the anchor hole; (**iv**) Metal deposition and patterning using a lift-off procedure; (**v**) SU-8 top insulation layer spin-coating and patterning of the probe outline and the anchor hole for the insertion of a 50 µm tungsten shuttle. Finally, the probes were released from the silicon substrate using buffered oxide etchant (1:7) in acid hood. (**b**) Optical picture of a GC MEA on SU-8 substrate with a metal interconnection and GC microelectrodes, after the release from the wafer. In inset is reported a different view of the MEA flexible shank fabricated with an anchor hole at the shank tip to facilitate the insertion of a 50 µm tungsten shuttle that enables the handling and penetration of the probes into the brain. (**c**) Flexible GC MEAs connected to the PCB using a zero-insertion force (ZIF) connector.

After cleaning, the wafer was spin-coated with an AZ P4620 photoresist (MicroChemicals) at 5300 rpm for 1 min and baked at 105 °C for 5 min. After soft baking, the wafer was exposed using MLA with a dose of 700 mJ/cm^2^, then developed using an AZ400k 1:4 developer (MicroChemicals), cleaned with water, rinsed, and dried by N2 gas flow. A 10 nm Ti adhesion layer and 100 nm Au layer were evaporated on the wafer using an electron-beam evaporator (Plassys MEB550S), and then the metal was lifted off in acetone to define the metal traces and connection pads. A top insulation layer of SU-8 2005 was then spin-coated at 4000 rpm for 1 min, soft based at 65 °C for 3 min and 95 °C for 5 min, and photolithography patterned, using MLA with a dose of 300 mJ/cm^2^, to expose the connection pads and to define the top insulation layer. After post-baking and a development procedure with the SU-8 developer, the wafer was cleaned with isopropanol and DI water, hard based at 200 °C, 180 °C, and 150 °C for 5 min each, and allowed to cool down below 95 °C. The MEAs were lifted off from the wafer using a buffered oxide etchant (1:7) in an acid hood for 4–6 h. An anchor hole was also patterned at the shank tip to facilitate the insertion of a 50 µm tungsten shuttle, to enable the handling and penetration of the flexible device into the brain. Figure 1a shows the schematic of the GC MEA fabrication.

### 2.2. PEDOT/CNT Coating and Electrochemical Characterization

Multiwalled CNTs (length of 10–30 μm and diameter of 20–30 nm, Cheap Tubes Inc. Brattleboro, VT, USA) were functionalized using our previously established methods [94,95]. Briefly, the CNTs were pretreated by dispersing 200 mg CNTs in 100 mL 1:3 concentrated HNO_3_ and H_2_SO_4_ solution with sonication for 2 h. The suspension was then kept at 30 °C overnight while stirring the solution. After the acid treatment, the CNTs were washed with water and separated using ultracentrifugation repeatedly until the pH of the washing solution was neutral. Finally, the CNTs were collected and dried at 60 °C.

PEDOT/CNT coatings were electropolymerized on the GC electrodes using a previously reported procedure [93,94,95]. Briefly, electropolymerization was carried out in an aqueous solution of 0.02 M 3,4-ethylenedioxythiophene (EDOT; Sigma Burlington, USA) containing 1 mg mL^−1^ functionalized CNT with a constant potential of 0.9 V until a 100 mC/cm^2^ charge deposition cut-off was reached.

Electrochemical impedance spectroscopy (EIS) measurements were used to investigate the electrode/solution interface before and after the PEDOT/CNT coating, quantify their impedance in the 1 Hz–100 kHz range [96] *in vitro*, and verify the functionality of the MEAs immediately after implantation, *in vivo*, as previously reported [93,97]. During the EIS measurements, a sine wave (10 mV RMS amplitude) was superimposed onto the open-circuit potential while varying the frequency from 1 to 10^5^ Hz.

Cyclic voltammetry (CV) was performed to quantify the capacitive charging of the GC microelectrodes before and after the PEDOT/CNT coatings. During the CV tests, the working-electrode potential was swept between 0.8 and −0.6 V vs. Ag/AgCl, maintaining a scan rate of 100 mV/s. In vitro EIS and CV were performed in 1× PBS in a three-electrode electrochemical cell set-up with a platinum counter electrode and an Ag/AgCl wire reference electrode. *In vivo*, a screw was used as the counter electrode, and the Ag/AgCl wire reference electrode was placed in contact with the brain through a small pinhole craniotomy.

Electropolymerization, EIS, and CV were carried out using a potentiostat/galvanostat (Autolab, Metrohm, Riverview, FL, USA).

### 2.3. SWV In Vitro Calibration

Electrochemical detection of DA was performed via SWV, similarly to our previous study [93]. SWV experiments were carried out using a potentiostat/galvanostat (AutoLab, Metrohm, Utrecht, The Netherlands) connected to a three-electrode electrochemical cell with a platinum counter electrode and an Ag/AgCl reference electrode. The SWV waveform was repeatedly applied from −0.2 V to 0.3 V with a 25 Hz step frequency, a 50 mV pulse amplitude, and a 5 mV step height every 15 s. The potential was held at 0 V between scans. In vitro DA calibrations were performed using freshly prepared DA solutions dissolved in aCSF (142 mM NaCl, 1.2 mM CaCl_2_, 2.7 mM KCl, 1.0 mM MgCl_2_, 2.0 mM NaH_2_PO_4_, pH 7.4) in a 50 nM^–1^ μM concentration range. Electrode sensitivity was determined by the slope of the linear range of the calibration plot relating the DA peak current at 0.7 V to the DA concentration.

### 2.4. FSCV In Vitro Calibration

Fast-scan cyclic voltammetry (FSCV) was performed with a 4-channel Wave Neuro potentiostat (Pine Research, United States) and collected and analyzed using HDCV software (University of North Carolina at Chapel Hill, NC, United States). The electrode was scanned using a triangular waveform with a negative holding potential of −0.4 V, a 1.3 V switching potential, and applied using a 400 V/s scan rate at 10 Hz. DA was identified by inspection of the background-subtracted cyclic voltammograms (DA oxidation peak occurred at 0.7 V). Electrodes were calibrated using 0.1–1 μM DA concentrations dissolved in aCSF. The different concentrations were diluted starting from freshly prepared 1 mM DA solutions. Calibration was performed in a flow cell equipped with a pneumatically actuated injection valve with a 500 μL sample loop (VICI AG International, Schenkon, Switzerland. Flow through the system at 60 mL/h was driven with a syringe pump, as previously reported [97,98].

### 2.5. In Vivo Procedures

*In vivo* performance was determined through acute and chronic surgical experiments conducted in the dorsal striatum (DS) of 8 male mice (C57BL/6J, 8–12 weeks, 22–35 g; Jackson Laboratory, Bar Harbor, ME, USA). All animal care and procedures were performed under approval of the University of Pittsburgh Institutional Animal Care and Use Committee and in accordance with regulations specified by the Division of Laboratory Animal Resources.

All animals were induced with 1.5–2% isoflurane mixed with oxygen flow at 1 L min^−1^, then maintained at 1.25–1.5%. Body temperature was maintained at 37 °C with a thermostatically controlled heating pad (Harvard Apparatus, Holliston, MA, USA), and Lacrigel (Dechra Puralube Vet Ointment) was placed on the eyes to avoid dryness.

After the animal head was fixed in a stereotaxic frame (Narishige International USA, Inc. Amityville, USA), the skin and connective tissue on the surface of the skull were removed. A small pinhole craniotomy was made over the DS (1 mm anterior to bregma, and 1.5 mm lateral from midline) with a high-speed dental drill (0.007 drill bit, Fine Science Tools, Inc., Foster City, CA, USA), and bone fragments were carefully removed with forceps and saline. Saline was applied continuously onto the skull to dissipate heat from the high-speed drill.

For SWV measurements, the GC MEAs or PEDOT/CNT-coated GC MEA were lowered 3.0 mm below the cortical surface into the DS using a micromanipulator. This resulted in five MEA electrode sites located in the DS. Two additional small pinhole craniotomies were performed for the introduction of the Ag/AgCl reference electrode contralaterally to the MEA and a bone screw counter electrode caudally to the reference (Supplementary Material Figure S1a). EIS was measured immediately after the MEA implantation. Then, the tonic DA response was measured using the SWV waveform (detailed above) over a 40 min period. To confirm the chemical specificity of our measurements, following 10 min of data collection, mice were administered with 2 mg/kg intraperitoneal (i.p.) raclopride, a selective antagonist on D2 dopamine receptors (Sigma Aldrich, St. Louis, MO, USA), and 20 mg/kg i.p. nomifensine, a dopamine reuptake inhibitor (Sigma Aldrich, St. Louis, MO, USA). In acute experiments, upon reaching the predetermined experimental endpoint, the MEAs were explanted, and the animals were humanely sacrificed using approved procedures.

For the SWV chronic experiment, after positioning the PEDOT/CNT-coated GC MEA, the craniotomy was filled with Kwik-Cast Sealant (World Precision Instruments, Sarasota, FL, USA), and dental cement (Pentron Clinical, Orange, CA, USA) was cured with a dental curing light to make a head cap. Immediately after, EIS was measured, and tonic DA response was measured using the SWV over a 5 min period. After surgery, animals were placed on an electric heating blanket under a warming light to wake up and received an i.p. injection of 5 mg/kg ketofen (100 mg/mL, Henry Schein) up to three days after. SWV detection was repeated every day for the first week and once a week for the subsequent weeks. SWV and EIS experiments were acquired using a potentiostat/galvanostat (Autolab PGSTAT128N, Metrohm, Utrecht, The Netherlands) connected to the three-electrode configuration: working electrode, bone screw (counter electrode), and Ag/AgCl wire reference electrode. DA peaks were isolated from the nonfaradaic background current for each SWV scan by subtracting a modeled polynomial baseline, using a previously described methodology [93]. *In vivo* DA concentration was determined for all *in vivo* experiments by converting the SWV peak current to the DA concentration using the pre-calibration electrode sensitivity, as previously reported [93].

A single proof-of-principle *in vivo* experiment was performed to evaluate the simultaneous multisite FSCV performance of GC MEAs. The GC MEAs were lowered 3.0 mm below the cortical surface into the DS using a hand-driven micromanipulator. An additional small pinhole craniotomy was performed for the introduction of the Ag/AgCl reference electrode contralaterally to the MEA. A second portion of skull and dura was removed for the introduction of a bipolar stainless-steel stimulating electrode (MS303/a; Plastics One, Roanoke, VA, USA), positioned over the medial forebrain bundle (MFB; the medial forebrain bundle 1.6 mm posterior to bregma, 1 mm lateral from bregma, and 4.8 mm below cortical surface) (Supplementary Information (SI) Figure S1b). To increase the DA phasic release concentration, the animal received an i.p. injection of a drug cocktail of 2 mg/kg raclopride (RAC), a D_2_ DA receptor antagonist, followed by 20 mg/kg nomifensine (NOM), an inhibitor of the dopamine transporter [99]. Ten minutes later, MFB stimulation was conducted via the application of an optically isolated stimulus waveform (Neurolog 800, Digitimer, Letchworth Garden City, UK) consisting of a biphasic, constant-current square wave (2 ms per pulse, 250 µA pulse height, 60 Hz frequency, 3 s duration) with the aforementioned bipolar stainless-steel electrode. Fast-scan cyclic voltammetry (FSCV) was performed with a 4-channel Wave Neuro potentiostat (Pine Research, Durham, NC, USA), and the data were collected and analyzed using HDCV software (University of North Carolina at Chapel Hill, NC, USA). The electrode was scanned using a triangular waveform with a negative holding potential of −0.4 V, a 1.3 V switching potential, and applied using a 400 V/s scan rate at 10 Hz.

## 3. Results and Discussion

### 3.1. GC MEA Fabrication

GC MEAs were successfully developed following the fabrication process schematically described in Figure 1a. Figure 1b shows an optical micrograph of a GC MEA on a SU-8 substrate with metal interconnections and GC microelectrodes, after the release from the wafer. In the inset is a different view of the flexible shank fabricated with an anchor hole at the tip to facilitate the insertion of a 50 µm tungsten shuttle that enables the handling and penetration of the probes into the brain. The device is composed of a singular shank (120 µm wide and ~15 µm thick) with 5 circular GC electrodes 40 µm in diameter. The total length of the shank is 5.5 mm to easily target the striatum of the mouse brain. Figure 1c shows how the flexible GC MEAs are connected to the PCB using a zero-insertion force (ZIF) connector, to be interfaced with characterization and recording systems.

Flexible SU-8 probes with a subcellular thickness dimension have been shown to provide seamless biointegration, improving the electrophysiological recording longevity [74,75,76]. The integration of carbon-based microelectrodes on a flexible SU-8 substrate will add electrochemical detection capabilities, offering multimodality together with the potential to achieve chronic implantation with a healthy and stable tissue interface.

### 3.2. FSCV Capability of GC MEAs

**Figure 2:**
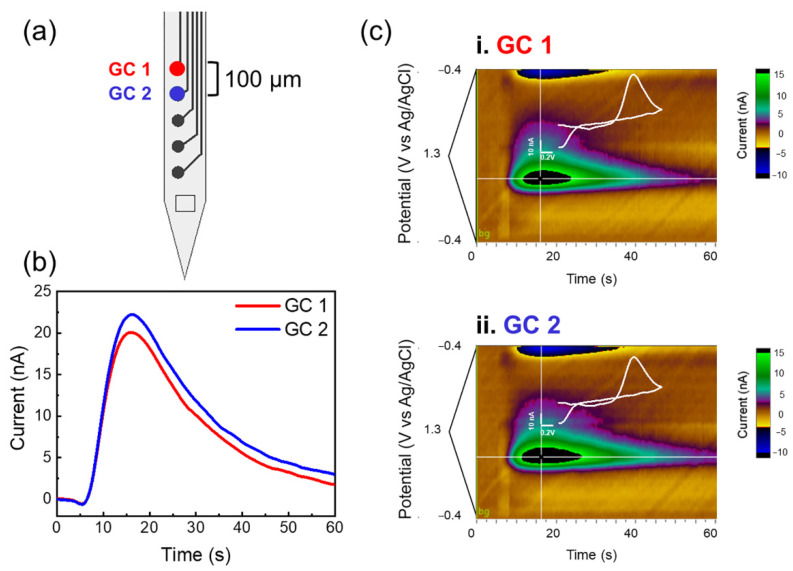
Multichannel detection from 2 adjacent GC microelectrodes (100 µm interelectrode distance). (**a**) Schematics of a 5-channel GC MEA with the electrode used pointed out. (**b**) Current/time plot of DA released in the dorsal striatum, evoked by electrical stimulation of DA axons in the medial forebrain bundle (MFB), using 2 adjacent GC microelectrodes; (**c**) corresponding color plots. These recordings are obtained after pharmacological manipulations (2 mg/kg raclopride and 20 mg/kg NOM).

**Figure 3:**
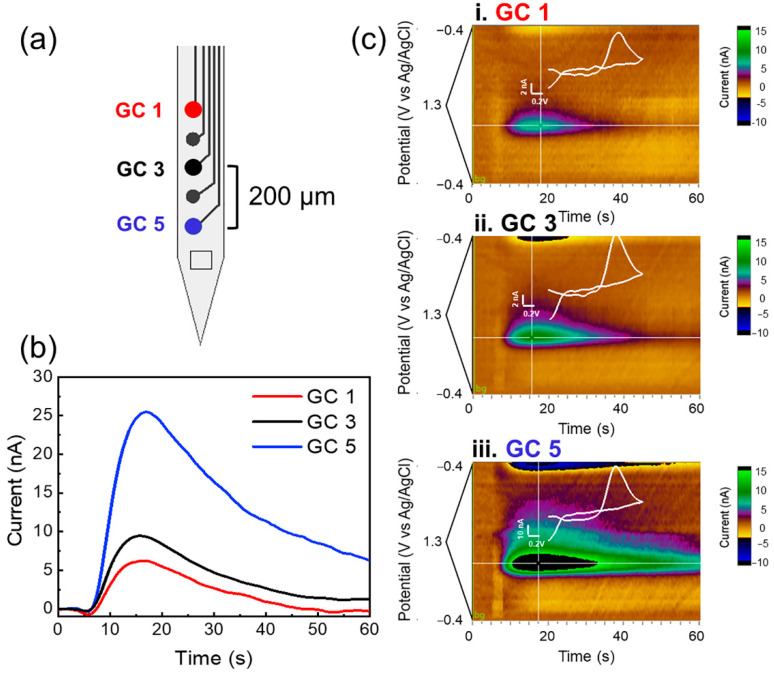
Multichannel detection from 3 GC channels (200 µm interelectrode distance). (**a**) Schematic of a 5-channel GC MEA with pointed out the electrode used. (**b**) Current/time plot of DA released in the dorsal striatum, evoked by electrical stimulation of DA axons in the medial forebrain bundle (MFB), using 3 GC microelectrodes with 200 µm interelectrode vertical distance; (**c**) corresponding color plots for evoked DA concentrations from the 3 different GC microelectrodes. These recordings are obtained after pharmacological manipulations (2 mg/kg raclopride and 20 mg/kg NOM).

Figure 2 presents an example of multichannel detection from two adjacent GC microelectrodes of the MEA, with a 100 µm interelectrode distance. The current/time plots and the corresponding color plots of the DA released in the DS, evoked by electrical stimulation of DA axons in the MFB, show very similar DA responses when detected from two adjacent GC microelectrodes. Interestingly, the evoked DA release detected from 3 alternate GC channels of the MEA, vertically spaced 200 µm center to center, show clear differences between channel responses, as shown by the current/time and color plots reported in Figure 3, highlighting the importance and necessity of multichannel detection to map the DA dynamics. Converting the current values into concentrations using the precalibration curve (Supplementary Information (SI) Figure S2a), we obtained an average concentration of 335.33 nM, in line with the values previously reported in the literature following similar pharmacological treatment [100,101]. An example of background-subtracted CV collected from a GC microelectrode in vitro, after 500 nM DA injection, and *in vivo*, after electrically stimulated DA release, is reported in Figure S2b of SI, showing similar oxidation peaks at the same potential versus Ag/AgCl. FSCV performance of GC microelectrodes for DA detection has been previously demonstrated [82,85]. GC microelectrodes have been shown to detect DA with high sensitivity, selectivity, and fouling resistance, being able to discriminate voltage reduction and oxidation peaks of DA and serotonin (5-HT) [85]. The high sensitivity of the GC microelectrodes has been attributed to the presence of curved graphene-like layers produced by the pyrolysis of SU-8 at 1000 °C, rich in hydroxyl, carbonyl, and carboxy functional groups [77,85]. These active groups and dense edge planes have been shown to be favorable for the adsorption of cationic species, such as dopamine, [85,102] and to increase hydrophilicity, which help to reduce fouling [77,102].

Here, we demonstrated that the newly fabricated GC MEAs on flexible SU-8 substrates with smaller dimensions are capable of detecting electrically stimulated DA release simultaneously at different locations of the DS from different GC microelectrodes on the same array. It is well known that there is anatomical and functional dopamine heterogeneity, and due to the lack of tools, it has been challenging to simultaneously sample DA at high spatial resolution to comprehensively understand DA functions [103,104,105]. By enabling simultaneous multisite detection of phasic DA release, our GC MEAS offers great potential in advancing neuroscience research of dopamine circuitry.

### 3.3. PEDOT/CNT Coating Enable Tonic DA Detection Using SWV

To integrate DA detection at different time resolutions on the same MEA, we coated GC microelectrodes with PEDOT/CNT to enable direct tonic DA measurement *in vivo* via SWV [93]. We previously observed that PEDOT/CNT coatings are essential for highly sensitive tonic DA detection because they combine a high effective surface area with a high content of incorporated negatively charged acid-functionalized CNTs, both facilitating DA adsorption [93]. We also previously demonstrated that our PEDOT/CNT, combined with an optimized SWV waveform, is selective among the most common neurochemical interferents, presenting a minimum sensitivity change in the presence of an interference cocktail consisting of 200 μM AA, 10 μM uric acid (UA), and 10 μM DOPAC.

**Figure 4:**
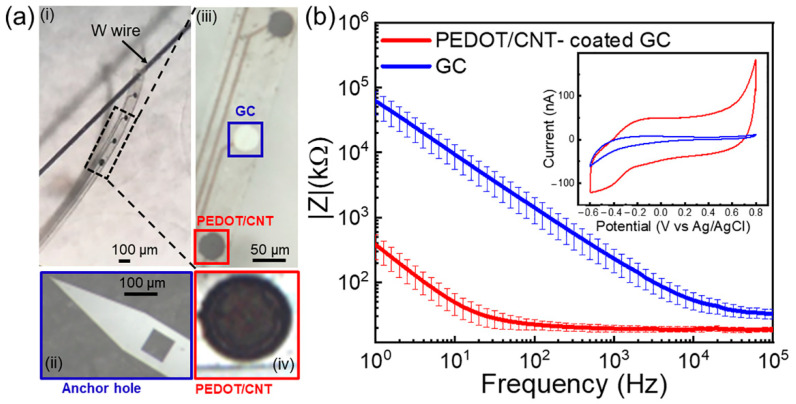
PEDOT/CNT-coated GC MEA. (**a**) (**i**) Optical picture of GC MEA on SU8 substrate with metal interconnection and GC microelectrodes, alternately coated with PEDOT/CNT. This prototype has been fabricated with an anchor hole at the shank tip to facilitate the insertion of a 50 µm W shuttle wire that will enable the handling and penetration of the probes into the brain (magnification in (**ii**)). (**iii**) Magnification on 3 electrodes, 2 coated and 1 uncoated GC in the center. (**iv**) Magnification on the PEDOT/CNT-coated GC microelectrodes. (**b**) Electrochemical Impedance Spectra of the magnitude impedance of PEDOT/CNT-coated (red) versus uncoated (blue) GC microelectrodes (mean and SD, n = 6) PEDOT/CNT coatings show more than one order of magnitude decrease over the frequency range of 1 Hz–100 kHz. In inset: representative example of a CV plot of a GC electrode, before (blue) and after (red) PEDOT/CNT coating.

We show here that the GC microelectrodes of 40 µm in diameter can be successfully coated with PEDOT/CNT and that the PEDOT/CNT coatings drastically decrease the impedance over all the measured frequency range (from 232.5 ± 51.1 kΩ to 19.4 ± 2.2 kΩ at 1 kHz, n = 6) and, in particular, in the low-frequency region (from 9.2 ± 3.8 MΩ to 48.8 ± 17.9 kΩ at 10 Hz, n = 6), where capacitance plays a significant role, due to the increase in the surface area [106,107] (Figure 4b). The CV plots (in inset) of the GC electrode, before (blue) and after (red) the PEDOT/CNT coating, confirmed an approximately 7× increased capacitance (Figure 4, inset). The charge storage capacity (CSC), calculated as the time integral of an entire CV cycle divided by the geometric area, increased from 12.57 ± 5.74 mC/cm^2^ to 87.28 ± 22.32 mC/cm^2^ after the PEDOT/CNT coatings. The sensitivity of PEDOT/CNT-coated GC MEAs for the electrochemical detection of tonic DA was first evaluated via in vitro calibration experiments performed in aCSF. We used an SWV waveform that we previously optimized by varying the parameters that can influence the voltammetry responses, i.e., frequency, step potential, pulse amplitudes, holding potential, and holding time [93]. The optimum waveform was determined to be a square wave with 50 mV pulse amplitude, 5 mV step height, and 25 Hz frequency, scanned from −0.2 to 0.3 V. To facilitate the diffusion of DA in and out of the porous PEDOT/CNT coatings, we demonstrated that it is effective to hold the potential at 0 V for 11 s between SWV repetitions [93].

**Figure 5:**
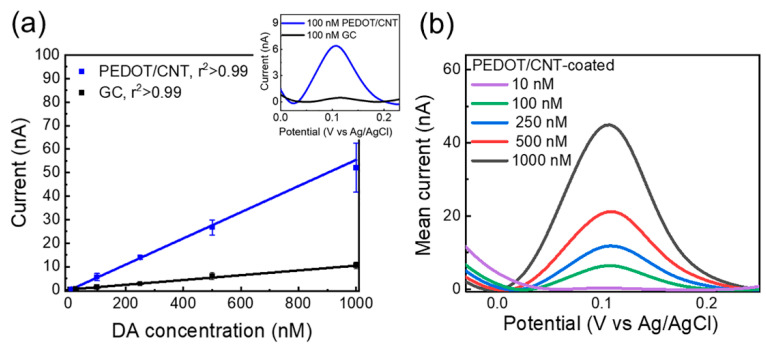
PEDOT/CNT-coated GC microelectrodes can detect basal DA concentration using SWV. (**a**) Average (±SD) DA calibration plot (peak current vs. DA concentration, n = 5) conducted at PEDOT/CNT-coated GC microelectrodes in comparison with the calibration plot obtained using GC microelectrodes (n = 4). The average sensitivity, defined as the linear slope of the calibration plot, is linear in the range of 10 nM–1 μM, and it is 5.6 times higher (55.634 ± 0.001 nA/μM) than the one obtained using uncoated GC microelectrodes (10.22 ± 0.33 nA/µM). In inset: representative baseline-subtracted SWV DA peaks collected from a PEDOT/CNT-coated microelectrode (blue) and GC microelectrodes (black). (**b**) In vitro SWV DA calibration conducted at PEDOT/CNT-coated GC microelectrodes in aCSF reveals clear DA peaks at 0.12 V.

Uncoated and PEDOT/CNT-coated GC microelectrodes of different MEAs were subjected to SWV measurement, first in aCSF, then in solutions of increasing DA concentration from 10 nM to 1 µM, designed to encompass the expected *in vivo* DA concentration range. PEDOT/CNT-coated GC microelectrodes exhibit clear DA detection at each concentration, with the average SWV traces revealing a single concentration-dependent peak located near 0.12 V (Figure 5a). Their response to DA is linear (r^2^ > 0.99, Figure 5b, blue) in the 10 nm–1 μM concentration range, with a 55.634 ± 0.001 nA/μM DA sensitivity, defined by the linear slope of the calibration plot relating the SWV peak current to the DA standard concentration. The average calibration plot for GC microelectrodes is also linear (r^2^ > 0.99, Figure 5b, black) but exhibits a DA sensitivity of 10.2 ± 0.3 nA/μM, approximately 6 times lower compared to the PEDOT/CNT-coated microelectrodes. Despite the sensitivity of the GC microelectrodes being higher than the previously reported sensitivity for carbon-fiber microelectrodes [93], the small peak amplitudes recorded at the low concentration range (<1 nA for 100 nM DA) make it difficult to clearly distinguish low DA concentrations (Figure 5a inset) using GC.

### 3.4. PEDOT/CNT-Coated GC MEAs Enable Multichannel Tonic DA Detection Using SWV

The *in vivo* capability of PEDOT/CNT-coated GC MEAs for multichannel detection of tonic DA level was first determined through acute surgical experiments conducted in the DS of isoflurane-anesthetized mice.

**Figure 6:**
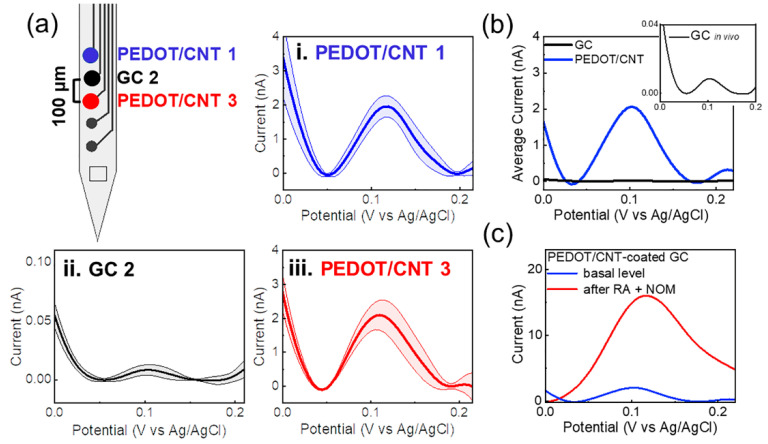
Multichannel detection of tonic dopamine concentrations using PEDOT/CNT-coated GC MEAs in the mouse DS. (**a**) Drawing of a 5-channel GC MEA with pointed out the electrode used for tonic DA detection, i.e., two PEDOT/CNT-coated microelectrodes (**i**,**iii**) and one uncoated GC microelectrode as a control (**ii**). (**i**,**iii**) Baseline-subtracted SWV DA peaks collected from two PEDOT/CNT-coated microelectrodes (basal level), (**ii**) baseline-subtracted SWV DA peaks collected from the uncoated GC microelectrodes; (**b**) baseline-subtracted SWV DA peaks collected at PEDOT/CNT-coated microelectrodes (blue) vs. GC microelectrodes (black, and magnification in inset); (**c**) representative baseline-subtracted SWV DA peaks collected at PEDOT/CNT-coated microelectrodes, before and 30 min after the administration of a cocktail of 2 mg/kg i.p. raclopride (RAC) and 20 mg/kg nomifensine (NOM).

The flexible MEAs were implanted into the DS using a 50 µm tungsten wire inserted into the anchor hole (Figure 1a and Figure 4a) and fixed with dissolvable PEG, which enabled the penetration of the flexible device into the striatum, resulting in five electrode sites located in the DS with a 100 µm interelectrode vertical distance. Immediately after implantation, the tungsten wire was removed, and the EIS was performed to ensure the proper electrode functionality (Figure S3, Supplementary Information). Then, the tonic DA response was measured using the SWV waveform detailed above over a 40 min period. Following 10 min of baseline data collection, mice were i.p. administered with a cocktail of 2 mg/kg i.p. RAC and 20 mg/kg NOM, known to selectively increase the DA concentrations [99], to confirm the chemical specificity of our measurements. Figure 6a reports an example of multichannel detection of tonic DA from two representative PEDOT/CNT microelectrodes (200 µm interelectrode vertical distance) and one adjacent uncoated GC microelectrode (signal averaged over 5 min of time collection for basal level). GC and PEDOT/CNT-coated microelectrodes are capable of tonic DA detection *in vivo*, as demonstrated by the SWV measurements, revealing a clear DA peak in the mouse dorsal striatum. PEDOT/CNT coatings drastically increased the sensitivity, detecting one order of magnitude higher peak amplitude than GC uncoated microelectrode (Figure 6a,b). The current peak drastically increased after pharmacological manipulation, reaching its maximum approximately after 30 min from the administration of the drug cocktail (Figure 6c). Converting the current values into concentrations using the precalibration curve, similar to a previous report [93], we estimate a DA basal level of 56.2 ± 12.3 nM, that increases up to 288.2 ± 24.6 nM after 30 min from drug administration (mean ± SD, n = 5).

The basal DA concentration measured in the DS using PEDOT/CNT-coated GC MEAs is slightly lower, but comparable, to previous measurements of basal DA level obtained using the same SWV techniques at PEDOT/CNT-coated CFEs in the rat dorsal striatum (82 ± 6 nM) [93]. These values are in line with the values obtained using other electrochemical techniques, such as FSCAV in the mouse nucleus accumbens of mice (90 ± 9 nM) [57] and convolution-based FSCV in the rat nucleus accumbens (41 ± 13 nM) [58].

**Figure 7:**
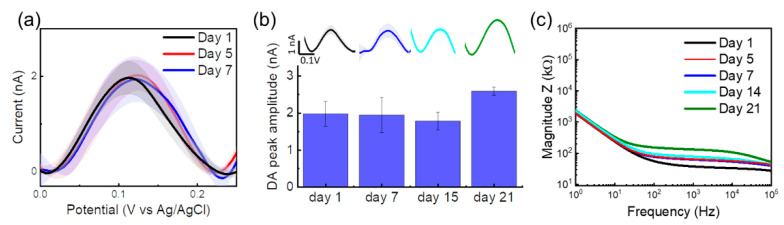
Stable chronic detection of tonic dopamine concentrations using PEDOT/CNT-coated GC MEAs in the mouse DS. (**a**) Baseline-subtracted SWV DA peaks collected from a PEDOT/CNT-coated microelectrode (basal level, mean, and STD of 5 min acquisition sessions) at Days 1, 5, and 7 post-implantation in the mouse DS. (**b**) Bar plot reporting the baseline-subtracted SWV DA peaks at 0.12 V, at Days 1–21 post-implantation in the mouse DS. Average ± SD of the SWV collected over a 5 min recording session, with the respective baseline-subtracted SWV DA peaks (top). (**c**) *In vivo* electrochemical impedance spectra of the magnitude impedance of a representative PEDOT/CNT-coated microelectrode implanted in the mouse DS at Days 1, 5, 7, 14, and 21 post-implantation.

Finally, as a proof of concept, we tested the stability of the DA tonic detection of our flexible MEA through a chronic experiment. We implanted a PEDOT/CNT-coated GC MEA in the mouse DS, as detailed above in the Materials and Methods (Section 2), and we tested the tonic DA SWV sensing performance over a 21-day period. The results are reported in Figure 7. The PEDOT/CNT-coated MEA shows an impressively stable tonic DA detection along the first week of implantation (Figure 7a), with DA peak amplitudes close to 2 nA (1.97 ± 0.34 Day 1 versus 1.94 ± 0.47 Day 7). We noticed a 0.08 V to 0.1 V peak shift starting from Day 14, likely caused by the dechlorination of the chronically implanted Ag/AgCl reference electrode [108], resulting in errors in the potential reading at the working electrode with consequent oxidation peak shifts [109,110]. However, the peak is still clearly detectable, and the peak amplitude remains stable at Days 14 and 21, with a 25% increase at Day 21 (Figure 7b), when we also observed a slight increase in resistivity in the impedance spectrum (Figure 7c).

Overall, we observed good electrode stability, with minimal variations in the peak amplitude of DA current and electrochemical impedance over the 21-day period (Figure 7). These results suggest that our flexible PEDOT/CNT-coated GC MEAs can enable stable chronic detection of tonic DA concentrations. The chronic stability of sensing could be the result of several factors: high stability of the PEDOT/CNT coating on GC, high electrochemical stability of the PEDOT/CNT and GC, high fouling resistance of CNT, and minimum glial inflammatory host tissue reaction. Electrochemically polymerized PEDOT doped with CNT has previously shown excellent stability in chronic recording [94] and stimulation [95] studies. Although detachment of PEDOT coatings from metal substrate has been identified as a major failure mode, doping PEDOT with CNT greatly improves the coating adhesion. Furthermore, the adhesion of PEDOT on GC electrodes has demonstrated superior adhesion to PEDOT on metal [80]. The combination of CNT doping and GC substrate should further enhance the adhesion. The antifouling resistance of the PEDOT/CNT interface may be due to the incorporation of the negatively charged acid-functionalized CNTs, similarly to what was previously observed for CNT, [79,111] CNT yarn [112], and other carbon nanomaterials, such as carbon nanohorns [113] and nanostructure graphene flakes [102]. Finally, the implant/tissue interface stability is promoted by the thin SU-8 flexible substrate that has previously been shown to trigger less foreign body response and promote seamless integration of the implanted device and brain tissue [75,76].

These promising results add incredible value to our technology because chronic sampling across multiple weeks is critical to the investigation of DA dynamic changes during neurological state transitions, for example from drug use to drug dependence in animal models of addiction, and to the understanding of the therapeutic effect of different medications.

## 4. Conclusions

This study presents the first GC MEA on flexible substrates for multichannel detection of both tonic and phasic DA concentrations *in vivo*, enabling DA detection at different time scales and multiple measurements within a microenvironment.

Using FSCV at GC microelectrodes, GC MEAs demonstrated multichannel simultaneous detection of phasic electrically stimulated DA release in the mouse DS. Using SWV at PEDOT/CNT-coated microelectrodes, the same MEAs enabled highly sensitive direct tonic DA measurement *in vivo* using SWV. Additionally, as a proof of concept, chronically implanted PEDOT/CNT-coated GC MEAs on a thin, flexible SU-8 substrate demonstrated stable tonic DA detection *in vivo* over a 3-week period.

Our results highlight the potential of flexible GC MEAs as a promising platform for integrated tonic and phasic multisite detection of DA, providing an unprecedented sensor for the study of the complex spatial and temporal pattern of DA dynamics in brain functions and dysfunctions. Such a platform can also offer electrophysiology functionality for multimodal brain mapping and closed-loop deep-brain stimulation.

## Data Availability

The data presented in this study are available on request from the corresponding author.

## Supplementary Materials

The following supporting information can be downloaded at: https://www.mdpi.com/article/10.3390/bios12070540/s1, Figure S1. Schematic of the *in vivo* experimental set-up for square-wave voltammetry (SWV) and fast-scan cyclic voltammetry (FSCV) measurements. Figure S2: GC microelectrodes can detect phasic DA concentration using FSCV; Figure S3: *In vivo* electrochemical impedance.

## References

[1] Ott T., Nieder A. (2019). Dopamine and cognitive control in prefrontal cortex. Trends Cogn. Sci..

[2] Nieoullon A. (2002). Dopamine and the regulation of cognition and attention. Prog. Neurobiol..

[3] Cools R. (2008). Role of Dopamine in the motivational and cognitive control of behavior. Neuroscience.

[4] Aristieta A., Gittis A. (2021). Distinct globus pallidus circuits regulate motor and cognitive functions. Trends Neurosci..

[5] Dunigan A.I., Roseberry A.G. (2022). Actions of feeding-related peptides on the mesolimbic dopamine system in regulation of natural and drug rewards. Addict. Neurosci..

[6] Wise R.A., Rompre P.-P. (1989). Brain dopamine and reward. Ann. Rev. Psychol..

[7] Panigrahi B., Martin K.A., Li Y., Graves A.R., Vollmer A., Olson L., Mensh B.D., Karpova A.Y., Dudman J.T. (2015). Dopamine is required for the neural representation and control of movement vigor. Cell.

[8] Barter J.W., Eli S., Elu D., Bartholomew R.A., Rossi M., Shoemaker C.T., Esalas-Meza D., Egaidis E., Yin H.H. (2015). Beyond reward prediction errors: The role of dopamine in movement kinematics. Front. Integr. Neurosci..

[9] Groves P.M. (1983). A theory of the functional organization of the neostriatum and the neostriatal control of voluntary movement. Brain Res. Rev..

[10] Grace A.A. (2016). Dysregulation of the dopamine system in the pathophysiology of schizophrenia and depression. Nat. Rev. Neurosci..

[11] Zhang L., Doyon W.M., Clark J.J., Phillips P., Dani J.A. (2009). Controls of tonic and phasic dopamine transmission in the dorsal and ventral striatum. Mol. Pharmacol..

[12] Grace A.A. (2000). The tonic/phasic model of dopamine system regulation and its implications for understanding alcohol and psychostimulant craving. Addiction.

[13] Grace A. (1991). Phasic versus tonic dopamine release and the modulation of dopamine system responsivity: A hypothesis for the etiology of schizophrenia. Neuroscience.

[14] Höglinger G.U., Rizk P., Muriel M.P., Duyckaerts C., Oertel W.H., Caillé I., Hirsch E.C. (2004). Dopamine depletion impairs precursor cell proliferation in Parkinson disease. Nat. Neurosci..

[15] Lotharius J., Brundin P. (2002). Pathogenesis of Parkinson’s disease: Dopamine, vesicles and α-synuclein. Nat. Rev. Neurosci..

[16] Howes O., Kapur S. (2009). The dopamine hypothesis of schizophrenia: Version III--The final common pathway. Schizophr. Bull..

[17] Seeman P. (1987). Dopamine receptors and the dopamine hypothesis of schizophrenia. Synapse.

[18] Di Chiara G.J.D. (1995). The role of dopamine in drug abuse viewed from the perspective of its role in motivation. Drug. Alcohol Depend..

[19] Botticelli L., Di Bonaventura E.M., Del Bello F., Giorgioni G., Piergentili A., Romano A., Quaglia W., Cifani C., Di Bonaventura M.V.M. (2020). Underlying susceptibility to eating disorders and drug abuse: Genetic and pharmacological aspects of dopamine D4 receptors. Nutrients.

[20] Wise R.A., Robble M.A.J. (2020). Dopamine and addiction. Annu. Rev. Psychol..

[21] Bello N.T., Hajnal A. (2010). Dopamine and binge eating behaviors. Pharmacol. Biochem. Behav..

[22] Frieling H., Römer K.D., Scholz S., Mittelbach F., Wilhelm J., De Zwaan M., Jacoby G.E., Kornhuber J., Hillemacher T., Bleich S. (2010). Epigenetic dysregulation of dopaminergic genes in eating disorders. Int. J. Eat. Disord..

[23] Volkow N.D., Wang G.-J., Maynard L., Jayne M., Fowler J.S., Zhu W., Logan J., Gatley S.J., Ding Y.-S., Wong C. (2003). Brain dopamine is associated with eating behaviors in humans. Int. J. Eat. Disord..

[24] Denys D., Zohar J., Westenberg H.G.M. (2004). The role of dopamine in obsessive-compulsive disorder: Preclinical and clinical evidence. J. Clin. Psychiatry.

[25] Koo M.-S., Kim E.-J., Roh D., Kim C.-H. (2010). Role of dopamine in the pathophysiology and treatment of obsessive–compulsive disorder. Expert Rev. Neurother..

[26] Denys D., van der Wee N., Janssen J., De Geus F., Westenberg H.G. (2004). Low level of dopaminergic D2 receptor binding in obsessive-compulsive disorder. Biol. Psychiatry.

[27] Grace A.A. (1995). The tonic/phasic model of dopamine system regulation: Its relevance for understanding how stimulant abuse can alter basal ganglia function. Drug Alcohol Depend..

[28] Budygin E.A., Bass C.E., Grinevich V.P., Deal A.L., Bonin K.D., Weiner J.L. (2020). Opposite consequences of tonic and phasic increases in accumbal dopamine on alcohol-seeking behavior. iScience.

[29] Buse J., Schoenefeld K., Münchau A., Roessner V. (2013). Neuromodulation in Tourette syndrome: Dopamine and beyond. Neurosci. Biobehav. Rev..

[30] Denys D., de Vries F., Cath D., Figee M., Vulink N., Veltman D.J., van der Doef T.F., Boellaard R., Westenberg H., van Balkom A. (2013). Dopaminergic activity in Tourette syndrome and obsessive-compulsive disorder. Eur. Neuropsychopharmacol..

[31] Marklund P., Larsson A., Elgh A., Linder J., Riklund K.A., Forsgren L., Nyberg L. (2009). Temporal dynamics of basal ganglia under-recruitment in Parkinson’s disease: Transient caudate abnormalities during updating of working memory. Brain.

[32] Rafi H., Zestos A.G. (2021). Review—Recent advances in FSCV detection of neurochemicals via waveform and carbon microelectrode modification. J. Electrochem. Soc..

[33] Robinson D., Venton B.J., Heien M.L., Wightman R.M. (2003). Detecting subsecond dopamine release with fast-scan cyclic voltammetry in vivo. Clin. Chem..

[34] Saylor R.A., Hersey M., West A., Buchanan A.M., Berger S.N., Nijhout H.F., Reed M.C., Best J., Hashemi P. (2019). In vivo hippocampal serotonin dynamics in male and female mice: Determining effects of acute Escitalopram using fast scan cyclic voltammetry. Front. Neurosci..

[35] Taylor I.M., Jaquins-Gerstl A., Sesack S.R., Michael A.C. (2012). Domain-dependent effects of DAT inhibition in the rat dorsal striatum. J. Neurochem..

[36] Taylor I.M., Nesbitt K.M., Walters S.H., Varner E.L., Shu Z., Bartlow K.M., Jaquins-Gerstl A.S., Michael A.C. (2015). Kinetic diversity of dopamine transmission in the dorsal striatum. J. Neurochem..

[37] Jacobs C.B., Ivanov I.N., Nguyen M.D., Zestos A.G., Venton B.J. (2014). High temporal resolution measurements of dopamine with carbon nanotube yarn microelectrodes. Anal. Chem..

[38] Keithley R.B., Takmakov P., Bucher E.S., Belle A.M., Owesson-White C.A., Park J., Wightman R.M. (2011). Higher sensitivity dopamine measurements with faster-scan cyclic voltammetry. Anal. Chem..

[39] Oh Y., Park C., Kim D.H., Shin H., Kang Y.M., DeWaele M., Lee J., Min H.-K., Blaha C.D., Bennet K.E. (2016). Monitoring in vivo changes in tonic extracellular dopamine level by charge-balancing multiple waveform fast-scan cyclic voltammetry. Anal. Chem..

[40] Wood K.M., Hashemi P. (2013). Fast-scan cyclic voltammetry analysis of dynamic serotonin reponses to acute escitalopram. ACS Chem. Neurosci..

[41] Swamy B.E.K., Venton B.J. (2007). Carbon nanotube-modified microelectrodes for simultaneous detection of dopamine and serotonin in vivo. Analyst.

[42] Meunier C.J., McCarty G.S., Sombers L.A. (2019). Drift subtraction for fast-scan cyclic voltammetry using double-waveform partial-least-squares regression. Anal. Chem..

[43] DeWaele M., Oh Y., Park C., Kang Y.M., Shin H., Blaha C.D., Bennet K.E., Kim I.Y., Lee K.H., Jang D.P. (2017). A baseline drift detrending technique for fast scan cyclic voltammetry. Analyst.

[44] Guida F., De Gregorio D., Palazzo E., Ricciardi F., Boccella S., Belardo C., Iannotta M., Infantino R., Formato F., Marabese I. (2020). Behavioral, biochemical and electrophysiological changes in spared nerve injury model of neuropathic pain. Int. J. Mol. Sci..

[45] Zestos A., Kennedy R.T. (2017). Microdialysis coupled with LC-MS/MS for in vivo neurochemical monitoring. AAPS J..

[46] Bungay P.M., Newton-Vinson P., Isele W., Garris P.A., Justice J.B. (2003). Microdialysis of dopamine interpreted with quantitative model incorporating probe implantation trauma. J. Neurochem..

[47] Gardner E.L., Chen J., Paredes W. (1993). Overview of chemical sampling techniques. J. Neurosci. Methods.

[48] Kennedy R.T., Watson C.J., Haskins W.E., Powell D.H., Strecker R.E. (2002). In vivo neurochemical monitoring by microdialysis and capillary separations. Curr. Opin. Chem. Biol..

[49] Krebs-Kraft D., Frantz K., Parent M., Lajtha A., Baker G. (2007). In Vivo Microdialysis: A Method for Sampling Extracellular Fluid in Discrete Brain Regions. Handbook of Neurochemistry and Molecular Neurobiology: Practical Neurochemistry Methods.

[50] Yang H., Peters J.L., Michael A.C. (2002). Coupled effects of mass transfer and uptake kinetics on in vivo microdialysis of dopamine. J. Neurochem..

[51] Yang H., Thompson A.B., McIntosh B.J., Altieri S.C., Andrews A.M. (2013). Physiologically relevant changes in serotonin resolved by fast microdialysis. ACS Chem. Neurosci..

[52] Chefer V.I., Thompson A.C., Zapata A., Shippenberg T.S. (2009). Overview of brain microdialysis. Curr. Protoc. Neurosci..

[53] Beyene A.G., Yang S.J., Landry M.P. (2019). Tools and trends for probing brain neurochemistry. J. Vac. Sci. Technol. A: Vacuum, Surfaces Films.

[54] Di Chiara G., Tanda G., Carboni E. (1996). Estimation of in-vivo neurotransmitter release by brain microdialysis: The issue of validity. Behav. Pharmacol..

[55] Jaquins-Gerstl A., Michael A.C. (2015). A review of the effects of FSCV and microdialysis measurements on dopamine release in the surrounding tissue. Analyst.

[56] Atcherley C.W., Laude N.D., Parent K.L., Heien M.L. (2013). Fast-scan controlled-adsorption voltammetry for the quantification of absolute concentrations and adsorption dynamics. Langmuir.

[57] Atcherley C.W., Wood K.M., Parent K.L., Hashemi P., Heien M.L. (2014). The coaction of tonic and phasic dopamine dynamics. Chem. Commun..

[58] Johnson J.A., Rodeberg N.T., Wightman R.M. (2018). Measurement of basal neurotransmitter levels using convolution-based nonfaradaic current removal. Anal. Chem..

[59] Schwerdt H.N., Zhang E., Kim M.J., Yoshida T., Stanwicks L., Amemori S., Dagdeviren H.E., Langer R., Cima M.J., Graybiel A.M. (2018). Cellular-scale probes enable stable chronic subsecond monitoring of dopamine neurochemicals in a rodent model. Commun. Biol..

[60] Schwerdt H.N., Shimazu H., Amemori K.-I., Amemori S., Tierney P.L., Gibson D.J., Hong S., Yoshida T., Langer R., Cima M.J. (2017). Long-term dopamine neurochemical monitoring in primates. Proc. Natl. Acad. Sci. USA.

[61] Obien M.E.J., Deligkaris K., Ebullmann T., Bakkum D.J., Frey U. (2015). Revealing neuronal function through microelectrode array recordings. Front. Neurosci..

[62] FeiLi D., Schuettler M., Doerge T., Kammer S., Hoffmann K.P., Stieglitz T. (2006). Flexible organic field effect transistors for biomedical microimplants using polyimide and parylene C as substrate and insulator layers. J. Micromech. Microeng..

[63] Rubehn B., Wolff S.B.E., Tovote P., Lüthi A., Stieglitz T. (2013). A polymer-based neural microimplant for optogenetic applications: Design and first in vivo study. Lab a Chip.

[64] Rutherford E.C., Pomerleau F., Huettl P., Strömberg I., Gerhardt G.A. (2007). Chronic second-by-second measures of l-glutamate in the central nervous system of freely moving rats. J. Neurochem..

[65] Edell D., Toi V., McNeil V., Clark L. (1992). Factors influencing the biocompatibility of insertable silicon microshafts in cerebral cortex. IEEE Trans. Biomed. Eng..

[66] Szarowski D., Andersen M., Retterer S., Spence A., Isaacson M., Craighead H., Turner J., Shain W. (2003). Brain responses to micro-machined silicon devices. Brain Res..

[67] Kozai T.D.Y., Jaquins-Gerstl A.S., Vazquez A.L., Michael A.C., Cui X.T. (2015). Brain tissue responses to neural implants impact signal sensitivity and intervention strategies. ACS Chem. Neurosci..

[68] Engstrom R.C., Wightman R.M., Kristensen E.W. (1988). Diffusional distortion in the monitoring of dynamic events. Anal. Chem..

[69] Kawagoe K., Garris P., Wiedemann D., Wightman R. (1992). Regulation of transient dopamine concentration gradients in the microenvironment surrounding nerve terminals in the rat striatum. Neuroscience.

[70] Schwerdt H.N., Kim M., Karasan E., Amemori S., Homma D., Shimazu H., Yoshida T., Langer R., Graybiel A.M., Cima M.J. Subcellular electrode arrays for multisite recording of dopamine in vivo. Proceedings of the 2017 IEEE 30th International Conference on Micro Electro Mechanical Systems (MEMS).

[71] Agorelius J., Tsanakalis F., Friberg A., Thorbergsson P.T., Pettersson L.M.E., Schouenborg J. (2015). An array of highly flexible electrodes with a tailored configuration locked by gelatin during implantation—Initial evaluation in cortex cerebri of awake rats. Front. Neurosci..

[72] Castagnola E., Maiolo L., Maggiolini E., Minotti A., Marrani M., Maita F., Pecora A., Angotzi G.N., Ansaldo A., Fadiga L. Ultra-flexible and brain-conformable micro-electrocorticography device with low impedance PEDOT-carbon nanotube coated microelectrodes. Proceedings of the 2013 6th International IEEE/EMBS Conference on Neural Engineering (NER).

[73] Nimbalkar S., Castagnola E., Balasubramani A., Scarpellini A., Samejima S., Khorasani A., Boissenin A., Thongpang S., Moritz C., Kassegne S. (2018). Ultra-capacitive carbon neural probe allows simultaneous long-term electrical stimulations and high-resolution neurotransmitter detection. Sci. Rep..

[74] Liu J. (2018). Syringe Injectable Electronics. Biomimetics Through Nanoelectronics.

[75] Luan L., Wei X., Zhao Z., Siegel J.J., Potnis O., Tuppen C.A., Lin S., Kazmi S., Fowler R.A., Holloway S. (2017). Ultraflexible nanoelectronic probes form reliable, glial scar–free neural integration. Sci. Adv..

[76] Zhao Z., Li X., He F., Wei X., Lin S., Xie C. (2019). Parallel, minimally-invasive implantation of ultra-flexible neural electrode arrays. J. Neural Eng..

[77] Puthongkham P., Venton B.J. (2019). Recent advances in fast-scan cyclic voltammetry. Analyst.

[78] Yang C., Denno M.E., Pyakurel P., Venton B.J. (2015). Recent trends in carbon nanomaterial-based electrochemical sensors for biomolecules: A review. Anal. Chim. Acta.

[79] McCreery R.L. (2008). Advanced carbon electrode materials for molecular electrochemistry. Chem. Rev..

[80] Vomero M., Castagnola E., Ciarpella F., Maggiolini E., Goshi N., Zucchini E., Carli S., Fadiga L., Kassegne S., Ricci D. (2017). Highly stable glassy carbon interfaces for long-term neural stimulation and low-noise recording of brain activity. Sci. Rep..

[81] Zachek M. (2010). Development of Carbon-MEMS Based Device for the In Vivo Electrochemical Detection of Neurotransmitter Fluctuations.

[82] Castagnola E., Vahidi N.W., Nimbalkar S., Rudraraju S., Thielk M., Zucchini E., Cea C., Carli S., Gentner T.Q., Ricci D. (2018). In vivo dopamine detection and single unit recordings using intracortical glassy carbon microelectrode arrays. MRS Adv..

[83] Kassegne S., Vomero M., van Niekerk P., Hirabayashi M. (2016). Glassy Carbon Microelectrodes for Neural Signal Sensing and Stimulation. Book 2 Carbon-The Next Silicon?-Applications.

[84] Devi M., Vomero M., Fuhrer E., Castagnola E., Gueli C., Nimbalkar S., Hirabayashi M., Kassegne S., Stieglitz T., Sharma S. (2021). Carbon-based neural electrodes: Promises and challenges. J. Neural Eng..

[85] Castagnola E., Thongpang S., Hirabayashi M., Nava G., Nimbalkar S., Nguyen T., Lara S., Oyawale A., Bunnell J., Moritz C. (2021). Glassy carbon microelectrode arrays enable voltage-peak separated simultaneous detection of dopamine and serotonin using fast scan cyclic voltammetry. Analyst.

[86] Ansaldo A., Castagnola E., Maggiolini E., Fadiga L., Ricci D. (2011). Superior electrochemical performance of carbon nanotubes directly grown on sharp microelectrodes. ACS Nano.

[87] Yang C., Jacobs C.B., Nguyen M.D., Ganesana M., Zestos A.G., Ivanov I.N., Puretzky A.A., Rouleau C.M., Geohegan D.B., Venton B.J. (2015). Carbon nanotubes grown on metal microelectrodes for the detection of dopamine. Anal. Chem..

[88] Kassegne S., Vomero M., Gavuglio R., Hirabayashi M., Özyilmaz E., Nguyen S., Rodriguez J., Özyilmaz E., Niekerk N., Khosla A. (2015). Electrical impedance, electrochemistry, mechanical stiffness, and hardness tunability in glassy carbon MEMS μECoG electrodes. Microelectron. Eng..

[89] Jurkiewicz K., Pawlyta M., Zygadło D., Chrobak D., Duber S., Wrzalik R., Ratuszna A., Burian A. (2017). Evolution of glassy carbon under heat treatment: Correlation structure–mechanical properties. J. Mater. Sci..

[90] Sharma S., Kumar C.S., Korvink J.G., Kübel C. (2018). Evolution of glassy carbon microstructure: In situ transmission electron microscopy of the pyrolysis process. Sci. Rep..

[91] Vomero M., van Niekerk P., Nguyen V., Gong N., Hirabayashi M., Cinopri A., Logan K., Moghadasi A., Varma P., Kassegne S. (2016). A novel pattern transfer technique for mounting glassy carbon microelectrodes on polymeric flexible substrates. J. Micromechanics Microengineering.

[92] Bard A.J., Faulkner L.R., White H.S. (2022). Electrochemical Methods: Fundamentals and Applications.

[93] Taylor I.M., Patel N.A., Freedman N.C., Castagnola E., Cui X.T. (2019). Direct in vivo electrochemical detection of resting dopamine using Poly (3, 4-ethylenedioxythiophene)/Carbon Nanotube functionalized microelectrodes. Anal. Chem..

[94] Kozai T.D.Y., Catt K., Du Z., Na K., Srivannavit O., Haque R.-U.M., Seymour J., Wise K.D., Yoon E., Cui X.T. (2015). Chronic *In vivo* evaluation of PEDOT/CNT for stable neural recordings. IEEE Trans. Biomed. Eng..

[95] Luo X., Weaver C.L., Zhou D.D., Greenberg R., Cui X.T. (2011). Highly stable carbon nanotube doped poly(3,4-ethylenedioxythiophene) for chronic neural stimulation. Biomaterials.

[96] Rose T., Robblee L. (1990). Electrical stimulation with Pt electrodes. VIII. Electrochemically safe charge injection limits with 0.2 ms pulses (neuronal application). IEEE Trans. Biomed. Eng..

[97] Castagnola E., Robbins E.M., Woeppel K.M., McGuier M., Golabchi A., Taylor I.M., Michael A.C., Cui X.T. (2020). Real-time fast scan cyclic voltammetry detection and quantification of exogenously administered melatonin in mice brain. Front. Bioeng. Biotechnol..

[98] Taylor I.M., Robbins E.M., Catt K.A., Cody P.A., Happe C.L., Cui X.T. (2017). Enhanced dopamine detection sensitivity by PEDOT/graphene oxide coating on in vivo carbon fiber electrodes. Biosens. Bioelectron..

[99] Walters S.H., Robbins E.M., Michael A.C. (2016). Kinetic diversity of striatal dopamine: Evidence from a novel protocol for voltammetry. ACS Chem. Neurosci..

[100] Robinson J.D., Howard C.D., Pastuzyn E.D., Byers D.L., Keefe K.A., Garris P.A. (2014). Methamphetamine-induced neurotoxicity disrupts pharmacologically evoked dopamine transients in the dorsomedial and dorsolateral striatum. Neurotox. Res..

[101] Wu Q., Reith M.E.A., Kuhar M.J., Carroll F.I., Garris P.A. (2001). Preferential increases in nucleus accumbens dopamine after systemic cocaine administration are caused by unique characteristics of dopamine neurotransmission. J. Neurosci..

[102] Castagnola E., Garg R., Rastogi S.K., Cohen-Karni T., Cui X.T. (2021). 3D fuzzy graphene microelectrode array for dopamine sensing at sub-cellular spatial resolution. Biosens. Bioelectron..

[103] Collins A.L., Saunders B.T. (2020). Heterogeneity in striatal dopamine circuits: Form and function in dynamic reward seeking. J. Neurosci. Res..

[104] Lammel S., Lim B.K., Malenka R.C. (2014). Reward and aversion in a heterogeneous midbrain dopamine system. Neuropharmacology.

[105] Mao Z., Davis R.L. (2009). Eight different types of dopaminergic neurons innervate the Drosophila mushroom body neuropil: Anatomical and physiological heterogeneity. Front. Neural Circuits.

[106] Orazem M.E., Tribollet B. (2008). Electrochemical Impedance Spectroscopy.

[107] Cogan S.F. (2008). Neural Stimulation and Recording Electrodes. Annu. Rev. Biomed. Eng..

[108] Seaton B.T., Heien M.L. (2021). Biocompatible reference electrodes to enhance chronic electrochemical signal fidelity in vivo. Anal. Bioanal. Chem..

[109] Robbins E.M., Castagnola E., Cui X.T. Accurate and Stable Chronic Voltammetric Measurements in the Brain Enabled by a Replaceable Subcutaneous Reference Electrode. https://ssrn.com/abstract=3985174.

[110] Seaton B.T., Hill D.F., Cowen S.L., Heien M.L. (2020). Mitigating the effects of electrode biofouling-induced impedance for improved long-term electrochemical measurements in vivo. Anal. Chem..

[111] Hanssen B., Siraj S., Wong D.K. (2016). Recent strategies to minimise fouling in electrochemical detection systems. Rev. Anal. Chem..

[112] Yang C., Trikantzopoulos E., Jacobs C.B., Venton B.J. (2017). Evaluation of carbon nanotube fiber microelectrodes for neurotransmitter detection: Correlation of electrochemical performance and surface properties. Anal. Chim. Acta.

[113] Puthongkham P., Yang C., Venton B.J. (2018). Carbon nanohorn-modified carbon fiber microelectrodes for dopamine detection. Electroanalysis.

