# Supplementary material for: Flexible Glassy Carbon Multielectrode Array for In Vivo Multisite Detection of Tonic and Phasic Dopamine Concentrations
Source: Biosensors (Basel). 2022 Jul 20;12(7):540. doi: 10.3390/bios12070540 (PMC9312827; doi:10.3390/bios12070540)
Supplement: Supplementary file 1 [file biosensors-12-00540-s001.zip › biosensors-1811033-supplementary.pdf]

Supplementary Information

Flexible Glassy Carbon Multielectrode Array for *In Vivo* Multisite Detection of Tonic and Phasic Dopamine Concentrations

Elisa Castagnola [1], Elaine M. Robbins [1], Bingchen Wu [1,2], May Yoon Pwint [1,2], Raghav Garg [3], Tzahi Cohen-Karni [3,4] and Xinyan Tracy Cui [1,2,5,*]

[1] Department of Bioengineering, University of Pittsburgh, Pittsburgh, PA 15260, USA; elc118@pitt.edu (E.C.); emr72@pitt.edu (E.M.R.); biw15@pitt.edu (B.W.); m.pwint@pitt.edu (M.Y.P.)
[2] Center for Neural Basis of Cognition, University of Pittsburgh, Pittsburgh, PA 15261, USA
[3] Department of Materials Science and Engineering, Carnegie Mellon University, Pittsburgh, PA 15213, USA; thenamegarg@gmail.com (R.G.); tzahi@andrew.cmu.edu (T.C.-K.)
[4] Department of Biomedical Engineering, Carnegie Mellon University, Pittsburgh, PA 15213, USA
[5] McGowan Institute for Regenerative Medicine, University of Pittsburgh, PA 15219, USA
* Correspondence: xic11@pitt.edu

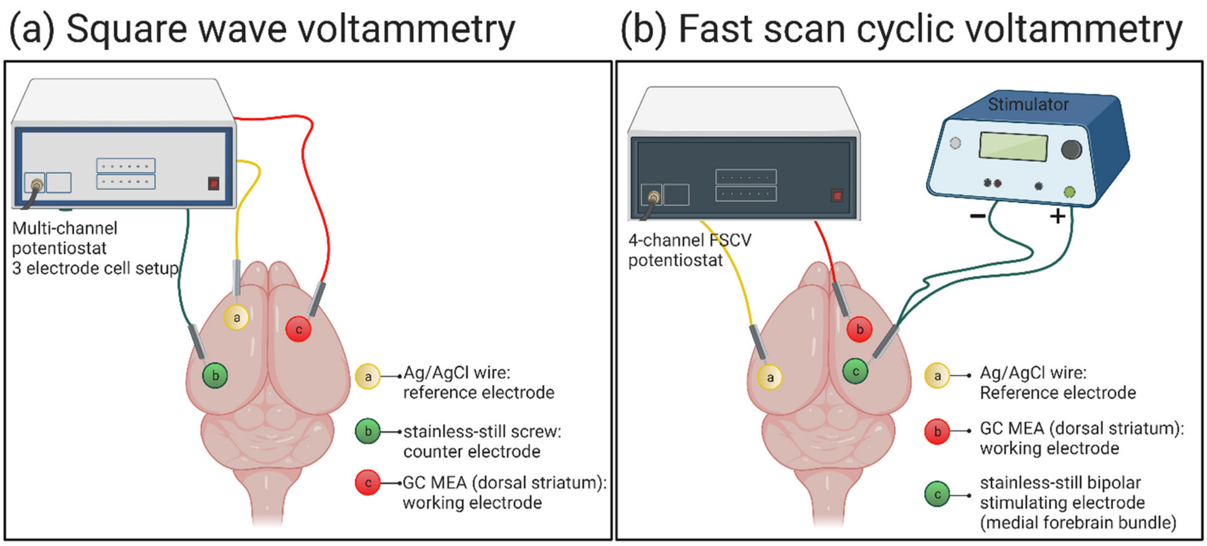

Figure S1. Schematic of the in vivo experimental setup for square wave voltammetry (SWV) and fast scan cyclic voltammetry (FSCV) measurements. Created with BioRender.com. (a) For SWV measurements, the GC MEAs or PEDOT/CNT coated GC MEA were lowered 3.0 mm below the cortical surface, into the dorsal striatum (DS) using a micromanipulator. This resulted in five MEA electrode sites located in the DS. Two additional small pinhole craniotomies were performed for the introduction of the Ag/AgCl reference electrode contralateral to the MEA and a bone screw counter electrode caudally to the reference. SWV experiments were acquired using a potentiostat/galvanostat (Autolab PGSTAT128N, Metrohm, USA) connected to in a three-electrode configuration: working electrode, bone screw (counter

electrode), and Ag/AgCl wire reference electrode. (b) For FSCV measurements, the GC MEAs were lowered 3.0 mm below the cortical surface, into the DS using a hand-driven micromanipulator. An additional small pinhole craniotomy was performed for the introduction of the Ag/AgCl reference electrode contralaterally to the MEA. A second portion of skull and dura were removed for the introduction of a bipolar stainless-steel stimulating electrode (MS303/a; Plastics One, Roanoke, VA, USA), positioned over the medial forebrain bundle (MFB; the medial forebrain bundle 1.6 mm posterior to bregma, 1 mm lateral from bregma and 4.8 mm below the cortical surface). MFB stimulation was conducted via the application of an optically isolated stimulus waveform (Neurolog 800, Digitimer, Letchworth Garden City, UK) consisting of a biphasic, constant-current square wave (2 ms per pulse, 250 μA pulse height, 60 Hz frequency, 3 second duration) with the aforementioned bipolar stainless-steel electrode. Fast scan cyclic voltammetry (FSCV) was performed with a 4 channel-Wave Neuro potentiostat (Pine Research, United States) and the data were collected and analyzed using HDCV software (University of North Carolina at Chapel Hill, NC, United States).

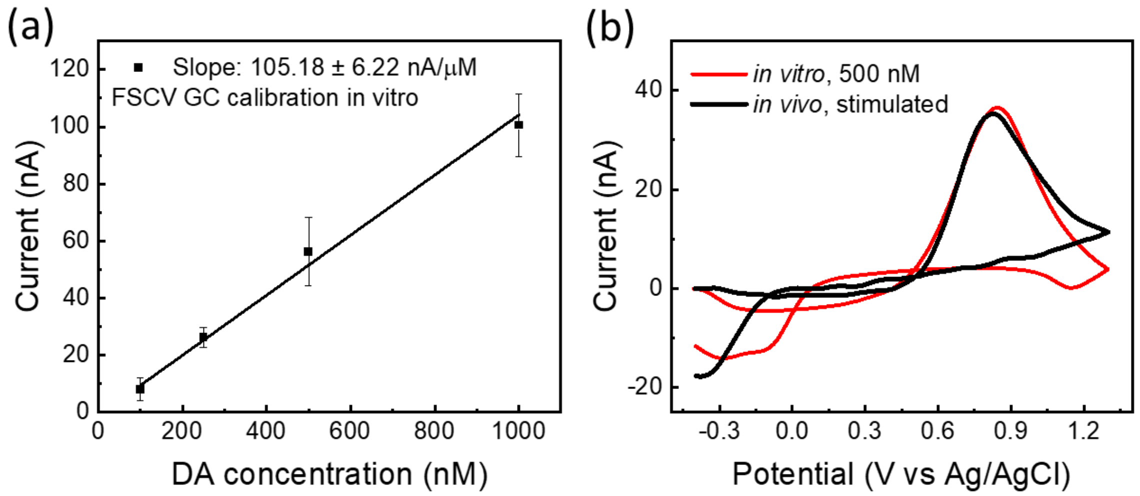

Figure S2: **GC microelectrodes can detect phasic DA concentration in using FSCV**. (a) Average (±SD) DA calibration plot (peak current vs. DA concentration, n=4) conducted at GC. The average sensitivity, defined as the linear slope of the calibration plot, is linear in the range 100 nM - 1 μM and it is 105.18±6.22 nA/μM. (b) Representative background-subtracted CV collected from a GC microelectrode in vitro, after 500nM DA injection, and in vivo, after electrically stimulated DA release in the dorsal striatum, show similar oxidation peaks.

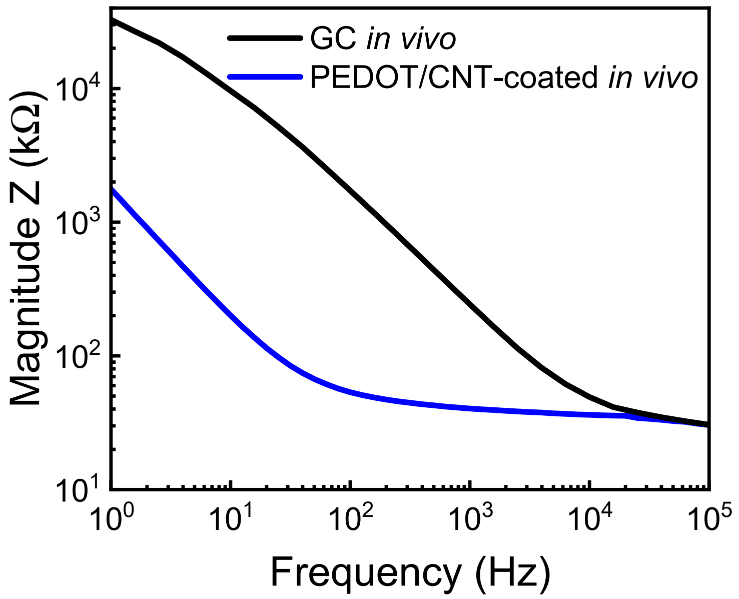

Figure S3: *In vivo* Electrochemical Impedance. *In vivo* electrochemical impedance spectra of the magnitude impedance of PEDOT/CNT coated microelectrodes versus GC uncoated microelectrodes implanted in the mouse DS immediately after implantation (n= 6).
